# On a Case of Resuscitation of a Still-Born Child

**Published:** 1855-10

**Authors:** John Wills

**Affiliations:** Jersey


*On a Case of Resuscitation of a Still-Born Child.* By JOHN WILLS, M. D., Jersey.—On the 8th inst. I was summoned to attend Mrs. J. with her first child, and after a natural labor of twelve hours she was safely delivered of a still-born child, and great was my disappointment at finding such to be the case, as Mrs. J. came from France here to be under my care.

How long life had ceased before the head was born I cannot say, but evidently life was quite extinct at birth, and the child's mouth and nose full of dark, sanious discharge, a large quantity of which passed with the child. There had been no liquor amnii.

After having divided the cord, I determined on trying to recover the child, and after having tried the usual means—shocks of cold water, &c.,—to no effect, I requested the nurse to bring me some hot water, into which I immersed it, the child then being quite cold, and sent to the husband to ask if he had a tube of any sort, but he had not; so I

procured a goose-quill, of which I made a tube, one end of which, guided by my finger, I passed into the larynx, and inflating the lungs with my breath, and emptying by pressure on the chest, which process I continued for more than half an hour, when I perceived a slight motion of the abdominal muscles, and, still persevering, my efforts were crowned with success, and I delivered the child to the nurse, crying lustily, much to the joy of the parents, as well as to my own satisfaction. The child has since done well.

This case, I think, fully proves that we ought always to make the attempt at resuscitation in still-born children, and it also proves by what simple means it may be brought about. Had I lost the child I should have blamed myself for having neglected taking with me, which I invariably do, an elastic catheter.—*London Lancet.*

